# *Globularia alypum L*. Modulates Inflammatory Markers in Human Colon and Shows a Potential Antioxidant Role in Myeloid Leukemic Cells

**DOI:** 10.37825/2239-9754.1030

**Published:** 2021-12-23

**Authors:** Najla Hajji, Elodie Hudik, Paola Iovino, Nacim Zouari, Hichem Sebai, Oliver Nusse, Carolina Ciacci

**Affiliations:** a Department of Medicine, Surgery and Dentistry, Scuola Medica Salernitana, University of Salerno, Fisciano, SA, Italy; b Université Paris-Sud and Université Paris-Saclay, Orsay, France; c Laboratoire de Physiologie Fonctionnelle et Valorisation des Bio-Ressources - Institut Supérieur de Biotechnologie de Béja, Université de Jendouba, Avenue Habib Bourguiba - B.P. 382 - 9000, Béja, Tunisia; d Higher Institute of Applied Biology of Medenine, Medenine, Gabes University, Tunisia; e CNRS, LCP, Orsay, France; f INSERM U1174, Orsay, France

**Keywords:** *Globularia alypum L*. aqueous extract, Oxidative stress, NF-κB, COX-2, Human colon biopsies, Inflammation

## Abstract

*Globularia alypum* (GA), a plant of the *Globulariacea* family, has long been used as a traditional cure for inflammatory and metabolic illnesses. In addition to various *in vitro* model studies, the current work focuses on the antioxidant and anti-inflammatory properties of GA in human colon biopsies.

The phenol components in GA aqueous extract (GAAE) were identified by Liquid Chromatography-Electrospray Ionization Mass Spectrometry. The antioxidant ability of GAAE was tested *in vitro* utilizing chemiluminescence and flow cytometry using fluorescent yeasts n conjunction with PLB-985-human myeloid leukemia cells. Experiments on human colon biopsies after a biopsy challenge with Escherichia coli-lipopolysaccharides aimed to see if GAAE had an anti-inflammatory impact on human colon inflammation. Western blotting was used to assess the expression of several inflammatory markers.

According to the findings, GAAE had a significant influence on hydrogen peroxide and cellular reactive oxygen species. GAAE inhibited the activities of cyclooxygenase 2 and nuclear factor B in inflamed biopsies, indicating anti-inflammatory action. The present study is the first to show that GA has a beneficial effect on human colon inflammation, thanks to its significant antioxidant activity *in vitro*. According to these preliminary data, GA may be utilized to treat a range of human inflammatory illnesses.

## 1. Introduction

All aerobic organisms create reactive oxygen species [1], which play a variety of essential activities. On the other hand, those species might cause possible harm to biomolecules, which can lead to the development of a variety of illnesses, including neurological disorders and cancer [2,3].

Oxidative stress is an imbalance between oxidants and antioxidants in favor of the oxidants [4]. Multiple repercussions of oxidating processes include lipid peroxidation, leading to membrane lipid degradation [5] and oxidative DNA damage [6], all of which contribute to cell viability loss. After being administered within several model systems, hydrogen peroxide (H_2_O_2_), a ROS, usually demonstrated cell damage or even cell death stimulation [7]. To date, the antioxidant medications used have made little or no impact on human health [8]. Potential data suggest that ROS and oxidative stress have a role in chronic and acute inflammation [9]. Infections and injuries cause inflammation, mediated by transcription factors such as NF-κB and STAT3, inflammatory enzymes such as cyclooxygenase 2 (COX-2), inflammatory cytokines such as tumor necrosis factor-alpha, and mitogen-activated protein kinases (MAPK) [10]. Both inflammation and oxidative stress are primary reasons implicated in chronic diseases and cancer, for which plants and phytochemicals are targeted to be safe cures [11].

*Globularia alypum L*. (GA), a member of the *Globulariaceae* family, is a common plant in the Mediterranean. GA has long been utilized in traditional medicine to treat various disorders caused by oxidative stress [12]. In his book “Medicinal Plants of North Africa,” Boulos (1983) listed GA as a medicinal plant used in traditional medicine in Morocco, Tunisia, Algeria, and Libya [13]. GA leaves were traditionally employed in Northeastern Morocco against diabetes, according to a published paper by Benjelloun (1997) regarding phytotherapy of hypertension and diabetes in Morocco and other physiological features of the plant such as laxative purgative, stomachic, and sudorific [12]. Numerous studies have shown that GA leaf and flower extracts are rich in secondary metabolites such as polyphenols and iridoids [14–16]. Those compounds are well known for their antioxidant properties [17], anti-inflammation [18], anti ulceration [19], and anticancer activities [20]. Several *in vitro* and *in vivo* studies have shown physiological activities of GA in oxidative stress regulation, which is the leading cause of those pathologies [15,21–23]. Moreover, GA has been shown for its anti-inflammatory effect *in vivo* against acetic acid-induced ulcerative colitis in rats [15].

In this study, the antioxidant impact of GA leaves aqueous extract (GAAE) was investigated *in vitro* utilizing flow cytometry and chemiluminescence techniques in both cellular and cell-free systems. We pioneered and implemented GAAE on biopsies taken from healthy patients with E. coli lipopolysaccharides, keeping in mind its anti-inflammatory properties against colitis in rats.

## 2. Materiel and methods

### 2.1. Plant collection and extraction of the aqueous extract

*Globularia alypum L*. (GA) was gathered in the northwestern part of Tunisia in March 2018. (Bou Salem, Jendouba). The Higher Institute of Biotechnology of Beja, University of Jendouba, has a voucher specimen with GA-ISBB-03-03-18. The GA leaves were recovered and dried for 72 hours at 40 °C in an incubator before being crushed in an electric blender (MOULINEX Ovatio 2. France). The resulting powder was dissolved in distilled water (10 g in 100 ml) and incubated at room temperature for 24 hours with agitation. After that, the sample was centrifuged at 10.000 g for 10 minutes, and the supernatant was lyophilized and kept at −80 °C for future use.

### 2.2. GAAE phenolic compounds by LC-ESI-MS

After filtering, a methanolic preparation was made from GAAE (1 g in 100 ml). The ultra-fast liquid chromatography column received one ml of the filtrated extract. An LCMS-2020 quadrupole mass spectrometer (Shimadzu, Kyoto, Japan) with an electrospray ionization source (ESI) and negative ionization mode was used for the LC-ESI-MS study. The mass spectrometer was connected to an LC-20AD XR binary pump system, SIL-20AC XR auto-sampler, CTO-20AC column oven, and DGU-20A 3R degasser through an ultra-fast liquid chromatography system (Shimadzu). The analysis was performed on an Aquasil C18 column (150 mm 3 mm, 3 m, Thermo Electron, Dreieich, Germany) preceded by an Aquasil C18 guard column (10 mm 3 mm, 3 m, Thermo Electron). The mobile phase A was 0.1% formic acid in H2O, and the mobile phase B was 0.1% formic acid in methanol, with a linear gradient elution of 0–45 minutes, 10–100% B, and 45–55 minutes, 100% B. Between individual runs, re-equilibration took 5 minutes. The mobile phase flow rate was 0.4 ml/min, with an injection volume of 5 μl. The column temperature was maintained at 40 °C. High-purity (>99%) nitrogen was employed as a nebulizer and auxiliary gas. Selected-ion monitoring (SIM) mode was used to monitor the spectra, and Shimadzu LabSolutions LC-MS software was used to analyze them. With a capillary voltage of 3.5 V, a dry gas flow rate of 12 μl/min, a nebulizing gas flow rate of 1.5 μl/min, a block source temperature of 400 °C, a dissolving line temperature of 250 °C, a voltage detector of 1.2 V, and full scan spectra from 50 to 2000 *m*/*z*, the mass spectrometer was operated in negative ion mode.

To identify GAAE compounds, the retention duration and mass spectra of phenolics were compared to those of genuine standards of highest purity (99.0%) (Sigma, Chemical Co. St Louis, MO, USA). p-coumaric acid, trans-ferulic acid, o-coumaric acid, trans-cinnamic acid, 4-o-caffeoylquinic acid, 1,3-di-o-caffeoylquinic acid, 3,4-di-o-caffeoylquinic acid, 4,5-di-o-caffeoylquinic acid, rosmarinic acid, salvianolic acid, catechin, epicatechin.

### 2.3. Effect of GAAE on L012-amped chemiluminescence in the presence of hydrogen peroxide (H_2_O_2_)

In the presence of horseradish peroxidase, the L012 (C13H2 CIN4NAO2, Waco) is oxidized by H_2_O_2_ (Sigma H1009) (HRP, Sigma P6782). A luminometer measured a light signal resulting from the process. With varying dosages of H_2_O_2_, different concentrations of the extract were tested. Five μl of L012 (10 μg/ml), 4 μl HRP (20 U/ml), 45 μl GAAE (25 ng/ml to 2.5 mg/ml), and 45 μl H_2_O_2_ (1 mM) were made for a total of 100 μl each, and the reaction was assessed for 30 minutes at 37 °C in the luminometer (Synergy H1 hybrid Reader Bioteck). The program Gen5 was used to examine the reaction kinetics.

### 2.4. DCFH2-A405-yeast preparation and flow cytometry

As is described by Tlili et al., double labeling heat-inactivated yeasts (Saccharomyces cerevisiae) with 2′,7′-dichlorodihydrofluorescein diacetate, succinimidyl ester (DCFH2, Invitrogen D399), and Alexa 405 was performed (Invitrogen A30000) [24]. The DCFH2 is oxidized to DCF in the presence of H_2_O_2_, resulting in a fluorescence signal activated by a blue laser (λ_ex_ 488 nm). Instead, Alexa 405 fluoresces with a purple laser (λ_ex_ 405 nm).

### 2.5. Flow cytometry analysis of the effect of GAAE against H_2_O_2_ on DCF-A405-yeast particles

DCFH2-A405 yeasts were incubated in the dark for 30 minutes with H_2_O_2_ (1 mM) and GAAE (25 ng/ml, 250 ng/ml, 2.5 μg/ml, and 25 μg/ml). At the same time, another batch of DCFH2-A405-yeasts was preoxidized for 30 minutes with H_2_O_2_ (1 mM). The yeasts were then rinsed in PBS to eliminate any leftover H_2_O_2_ and treated with the same GAAE doses for 30 minutes. A dual laser flow cytometer (405 nm and 488 nm) was used to detect the fluorescence intensity (Cyflow ML, Sysmex Partec, Germany). Summit V4.3.01 was used to evaluate the data (DAKO Colorado, Fort Collins CO, USA). The extract completely impacted DCFH2-fluorescence when it was co-incubated with H_2_O_2_. The quenching of DCF-fluorescence by GAAE was measured in a pre-oxidation experiment. The difference between the two experiments (co-incubation and pre-oxidation) determines the H_2_O_2_ scavenging activity by GAAE.

### 2.6. Cell culture

PLB-985 cells (human myeloid leukemia cell line) were grown at 37 °C in a 5% CO2 incubator in RPMI 1640 media supplemented with 10% fetal calf serum (FCS; heat-inactivated), l-glutamine (2 mM), penicillin (100 U/ml), streptomycin (100 μg/ml), and amphotericin B (0.25 g/ml) (Panasonic incu Safe). A cell culture passage was performed twice a week to keep the cell density between 2105 and 2106/ml. The DMSO (1.25%) (Sigma-Aldrich USA) was given to PLB-985 cells before studies and for 6 days to ensure that they differentiated into neutrophil-like cells [25].

### 2.7. GAAE effect in generating reactive oxygen species in PLB-985 cells

PLB-985 cells were pre-incubated with varied GAAE doses for 3 hours after differentiation. The cellular suspensions were then rinsed in HEPES buffer (140 mM NaCl, 5 mM KCl, 1 mM MgCl2, 2 mM CaCl2, 10 mM HEPES, pH 7.4) before being charged for 30 minutes at 37 °C with DCFH2-DA (2.5 μM). After a second wash, the cells were stimulated for 1 h with 200 nM PMA (Phorbol 12-myristate 13-acetate). Flow cytometry was then used to determine DCF fluorescence.

### 2.8. Collection and culture of biopsies

After receiving informed consent, data and biopsies were gathered from ten healthy participants who had a colonoscopy but found no pathology.

The colon biopsy culture is an *ex-vivo in vitro* model that can accurately duplicate the intestinal state, including all cell populations and relationships. Each biopsy was put above a stainless steel mesh in the center well of an organ culture dish (Falcon, USA), with the villous surface of the biopsies on top. To reach the biopsy’s cutting surface, the wells were filled with DMEM F12 (16 ml), fetal bovine serum (3 ml), penicillin 50,000 IU, and streptomycin 5000 IU culture media. The dishes were incubated at 37 °C in a sterile anaerobic jar gassed with oxygen/carbon dioxide (95/5%) [26].

Each patient’s biopsies were incubated separately in wells, with the first serving as a negative control containing just culture media, followed by four biopsies co-treated with GAAE (50 and 100 μg/ml) and EC-LPS (1 μg/ml), and the fifth serving as a positive control of inflammation with EC-LPS. After that, the cultures were permitted to incubate overnight. All cultures were halted after 24 hours, and biopsies were recovered and stored at −80 °C for future analyses.

### 2.9. Western blot

Colon samples were lysed in RIPA buffer for cytoplasmic protein extraction. Before the lyse, the buffer was treated with protease and phosphatase inhibitors, and then nuclear extraction buffer was added to the pellets to get nuclear proteins. Protein concentration was computed using a standard range of BSA from known values and quantified using the Bradford (Biorad) technique. 40 μg of total extracted proteins were adjusted with lysis buffer (RIPA), combined with LAEMMLI buffer (10 μl), and denatured for 5 min at 95 °C from total extracted proteins. The lysates were electrophoresed on a 10% sodium dodecyl sulfate/polyacrylamide gel (SDS-PAGE). The migration took 20 minutes at 80 V and then 120 V. Following the migration, proteins were transferred to a nitrocellulose membrane using a Biorad transfer cassette and incubated for 1 hour at room temperature with a blocking solution (5% dehydrated milk powder dissolved in TBST). Anti-COX-2 monoclonal antibody (1:1000, CAYMAN chemical business), anti–NF–κB (1:1000, Bioss Antibodies), anti-MAPK p38 (1:1000, Bioss Antibodies), and anti-ICAM-1 monoclonal antibody (1:1000, Bioss Antibodies) were all applied to the membranes individually overnight at 4 °C (1:1000, Santa Cruz Biotechnology). Membranes were incubated for 1 hour with horseradish peroxidase-linked goat anti-mouse or anti-rabbit secondary antibody (1: 1000, Immuno & Reagents) after three washes with TBST buffer. The rabbit antibody -actin and Lamin A/C were used to control the loaded protein quantification (1: 1000, ABCAM). The findings of Western blot detection were seen by chemiluminescence with the Chemidoc utilizing substrate reagents (Clarity Western ECL substrate, Biorad) (Bio-rad).

## 3. Results

### 3.1. GAAE phenolic compounds detection by Liquid Chromatography-Electrospray Ionization Mass Spectrometry (LC-ESI-MS)

The GAAE phenolic acid and flavonoids components were detected using LC-ESI-MS. As shown in Table 1, a total of 19 compounds were found. The most prevalent component identified (14720.4 ppm) was trans-cinnamic acid (also known as 3-phenyl-prop-2-enoic acid), which accounted for 72.99% of the whole extract, followed by quinic acid (13.98%) (Table 1). Luteolin 7-O-glucoside (5.73%), the extract’s third significant component, belongs to flavones, the extract’s second major class, along with apigenin, cirsiliol, and luteolin. Flavonoid molecules flavonol (quercetin and kaempferol) and favonone (naringin and naringenin) have also been identified.

### 3.2. GAAE scavenging activity of H_2_O_2_ on L012-amplified chemiluminescence

**Fig. 1:**
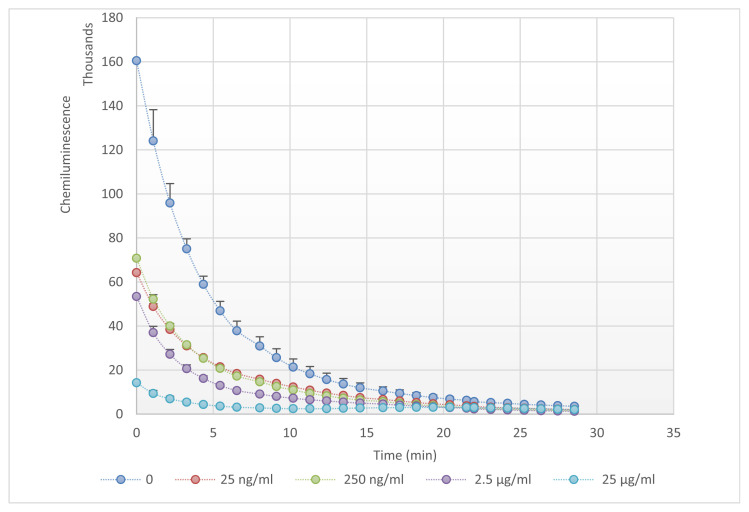
GAAE scavenging effect against H_2_O_2_ on L012-amplified chemiluminescence. GAAE (25 ng/ml; 250 ng/ml; 2.5 μg/ml and 25 ng/ml) incubated with 1 mMH_2_O_2_ (added at time 0) in the presence of HRP and L012 and luminescence measured during 30 min. The experiment was repeated 3 times, and results were represented as mean ± SEM.

Over 30 minutes, the GAAE potential to neutralize H_2_O_2_ was measured using L012-enhanced chemiluminescence in the presence of HRP. The H_2_O_2_ alone showed an exponential reduction in luminescence emission (Fig. 1). Even the lowest dose of GAAE(25ng/ ml) significantly diminished the signal when it was added. The signal was nearly totally suppressed in the presence of the highest concentration, indicating that GAAE has a substantial H_2_O_2_ scavenging action.

### 3.3. Evaluation of GAAE activity on H_2_O_2_ within DCF-A405-yeast system

**Fig. 2:**
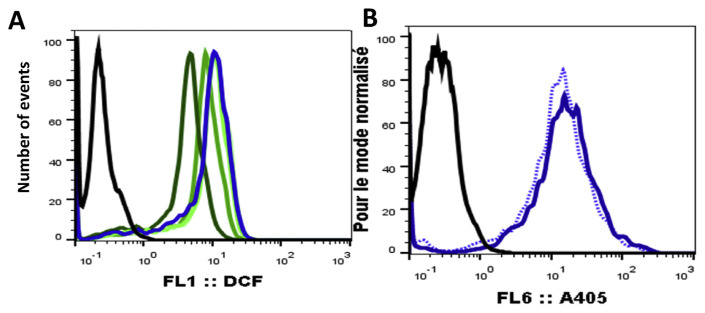
Effect of GAAE on DCFH2 & Alexa405 fluorescence. DCFH2 and Alexa 405 yeast were incubated with GAAE (25 ng/ml; 250 ng/ml; 2,5 μg/ ml and 25 μg/ml) and 1 mM H_2_O_2_ for 30 minutes. Flow cytometry was used to examine yeast fluorescence with an excitation wavelength of 488 nm (A): DCF fluorescence of non-oxidized DCFH2-labeled yeast; blue line: DCFH2-labeled yeast fully oxidized by H_2_O_2_, green lines: DCFH2-labeled yeast oxidized by H_2_O_2_, in the presence of GAAE, the dark green line represents the highest GAAE concentration (25 μg/ml), and results were represented as fluorescence histograms at 405 nm (B). The black line represents non-labeled yeast, the blue line represents Alexa 405-labeled yeast, and the dotted blue line represents Alexa 405-labeled yeast following incubation with 25 μg/ml GAAE.

In the presence and absence of GAAE, the tagged yeasts were oxidized by H_2_O_2_. The median fluorescence of each particle was used to estimate the fluorescence created by oxidized yeasts (Fig. 2 (A)). The GAAE reduced median fluorescence dose-dependent, indicating a potent anti-H_2_O_2_ action. However, in the presence of GAAE, Alexa 405, which exhibited a steady fluorescence in H_2_O_2_, experienced a minor reduction in fluorescence (Fig. 2 (B)). A signal dampening impact of DCF fluorescence by GAAE must be ruled out to extract colors with a broad absorption spectrum. Furthermore, the rapid response toward H_2_O_2_ on L012-amplified chemiluminescence (Fig. 1) might be owing to ROS scavenging, but it could also be due to the GAAE signal quenching on both fluorescence and luminescence. We examined the DCFH2 tagged yeast fluorescence in the presence of H_2_O_2_ and GAAE to its fluorescence when pre-oxidized by H_2_O_2_ to DCF, washed, and then incubated with GAAE to distinguish between scavenging and quenching effects.

**Fig. 3:**
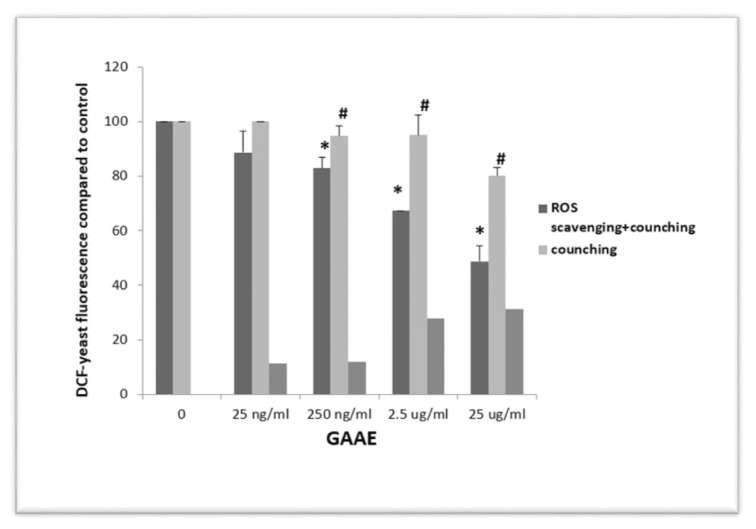
Distinction between ROS scavenging and signal quenching of GAAE against DCF-yeast. Flow cytometry data from DCFH2-A405 yeast were analyzed quantitatively. The first column on the left (dark grey) represents DCFH2-A405 yeast incubated with GAAE (25 ng/ml, 250 ng/ml, 2.5 μg/ml, and 25 μg/ml) and H_2_O_2_ for 30 minutes for each condition (1 mM). The second column (light grey) shows DCFH2-A405 yeast that has been pre-oxidized with H_2_O_2_ (1 mM), washed, and incubated with GAAE (25 ng/ml, 250 ng/ml, 2.5 μg/ml, and 25 μg/ml) for 30 minutes. This circumstance determines the quenching of DCF fluorescence by GAAE. The third column (medium grey) shows the difference between the two initial columns defining ROS scavenging activity is shown by the third column (medium grey). The median fluorescence of the results is normalized to the positive control (oxidized DCF-yeast without extract). The experiment was performed three times with the same findings. *p 0.05 compared to the positive control and #p 0.05 compared to the scavenging + quenching impact of each GAAE dose (Student-test).

As seen in Fig. 3, the extract suppressed DCF fluorescence dose-dependent. GAAE also reduced pre-oxidized yeast fluorescence, though to a lesser extent than in the co-incubation experiment (H_2_O_2_ and GAAE simultaneously).

At 25 ng/ml, the difference in fluorescence levels was not significant, but at the other three doses (Fig. 3). As a result, GAAE functions as a legitimate H_2_O_2_ scavenger.

### 3.4. Effect of GAAE against ROS production by PLB-985 cells

**Fig. 4:**
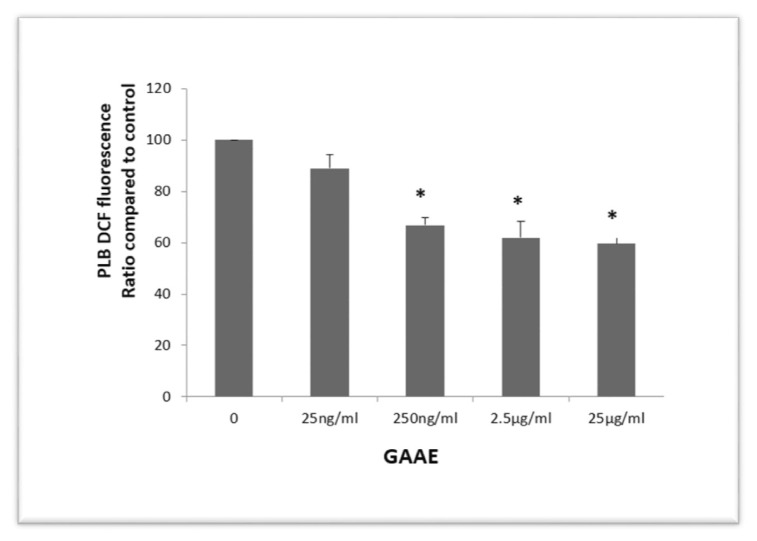
Pre-incubation effect of GAAE on ROS production by PLB-985 cells. LB-985 cells were pre-incubated for 3 hours with GAAE (25 ng/ml, 250 ng/ml, 2.5 μg/ml, and 25 μg/ml), then charged with H2DCF-DA (2.5 μM) and stimulated with PMA (200 nM). Flow cytometry was used to examine DCF fluorescence in PLB-985 cells, using excitation at 488 nm and findings expressed as median fluorescence adjusted to the positive control (stimulated PLB cells not treated with the extract). The experiment was conducted three times, with the data provided as mean SEM. *p 0.05 when compared to the positive control (Student-test).

The NADPH oxidase in neutrophils and macrophages produces a lot of ROS. ROS generation is essential for the immune response to infections because it helps to destroy pathogens following phagocytosis. Chronic inflammation is caused by immune cells producing too many reactive oxygen species. PLB-985 neutrophil-like cells are a helpful cell culture model for neutrophils and neutrophil functions research. In PLB-985 cells charged with DCFH2 and activated by PMA, the effect of GAAE on cellular ROS generation was investigated. PLB cells create ROS in the extracellular environment under these conditions. Some ROS make their way inside the cell and oxidize DCFH2 to DCF. Pre-treating PLB cells with GAAE for 3 h reduced DCF fluorescence in a dose-dependent manner, indicating an influence on the cellular capacity to create ROS or the PLB cells’ intracellular antioxidant capacity (Fig. 4). The cytotoxicity of the GAAE treatment was not revealed by staining the cells with propidium iodide.

### 3.5. GAAE decreases the EC-LPS effect on colon proinflammatory proteins

**Fig. 5:**
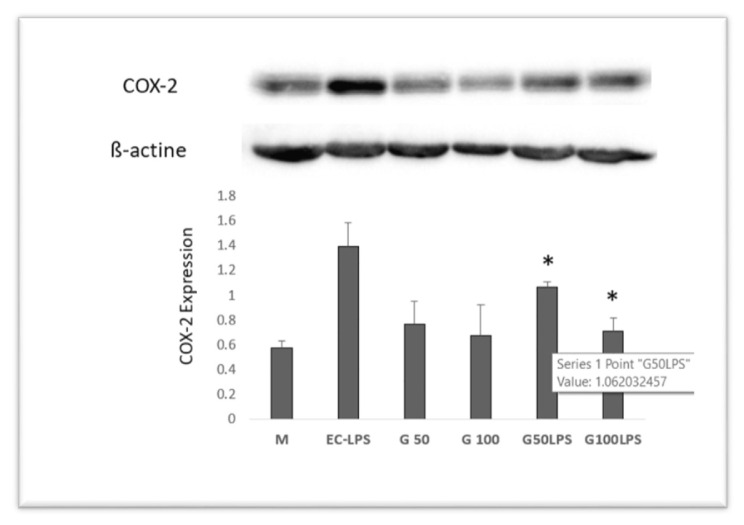
Effect of GAAE and EC-LPS co-treatment on COX-2 expression in human colon biopsy culture. Two GAAE dosages (50 and 100 μg/ml) and EC-LPS (1 μg/ml) were used to culture colon biopsies. Western blotting was used to measure COX-2 activity. The media of three distinct cultures from three different people are displayed as mean SEM.*p 0.05 compared to EC-LPS (Student-test).

To begin, GAAE was evaluated in biopsies from three healthy persons in co-culture with EC-LPS at two different concentrations (50 and 100 μg/ml) to determine its reaction for COX-2 expression. The results reveal that EC-LPS significantly boosted COX-2 expression (Fig. 5).

The addition of GAAE in the presence of EC-LPS dramatically lowered COX-2 expression, and the dose of 100 μg/ml was more efficient than the negative control in which the biopsies were cultured merely with the medium.

**Fig. 6:**
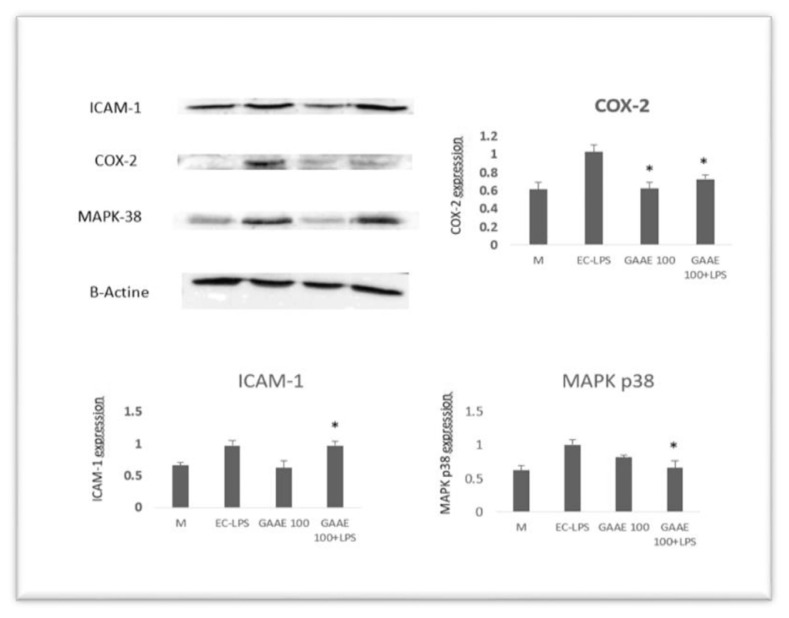
GAAE and EC-LPS co-treatment on proinflammatory marker expression in human colon biopsy culture. In the presence of a single GAAE dosage (100 g/ml) and EC-LPS (1 μg/ml), colon biopsies were cultured. Western blotting was used to measure the activities of COX-2, ICAM-1, and MAPK p38. The media of seven distinct cultures from seven different people are displayed as mean SEM.*p 0.05 compared to EC-LPS (Student-test).

**Fig. 7:**
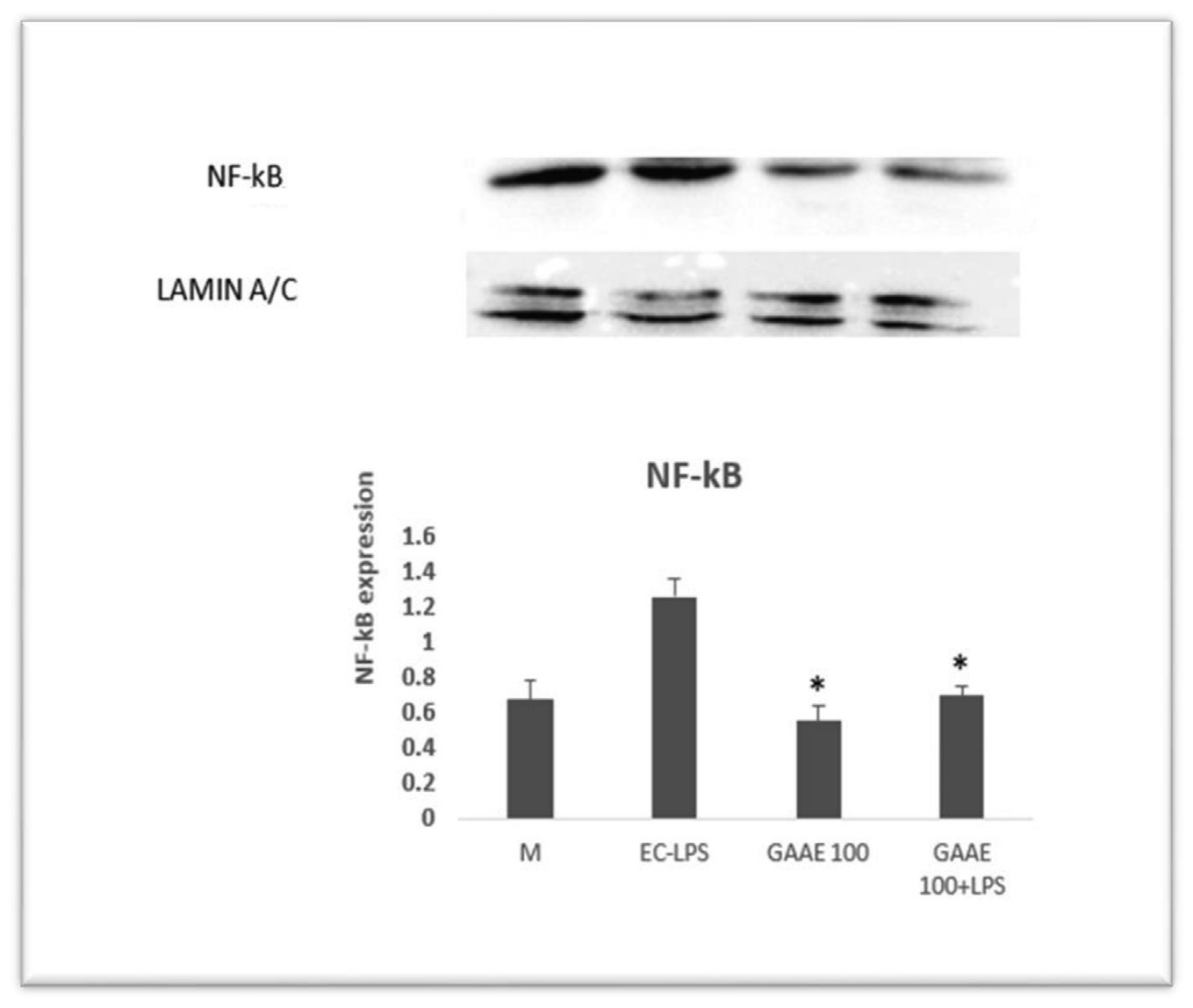
Effect of GAAE and EC-LPS co-treatment on nuclear NF-κB activity in human colon biopsy culture. In the presence of a single GAAE dosage (100 μg/ml) and EC-LPS (1 μg/ml), colon biopsies were cultured. Western blotting was used to measure the activity of nuclear NF-κB. The media of seven distinct cultures from seven different people are displayed as mean SEM.*p 0.05 compared to EC-LPS (Student-test).

The dose of 100 μg/ml was chosen to continue the investigation after that. The effects of GAAE and EC-LPS on COX-2, ICAM-1, MAPK p38 expressions (Fig. 6), and nuclear NF-κB activity were examined in biopsy cultures of seven additional healthy persons (Fig. 7). EC-LPS raised the expression of all proinflammatory markers in the majority of individuals. GAAE impacted the expression of those makers, with a more substantial effect on COX-2 and NF-κB than ICAM-1 and MAPK p38, where responses were not always positive or significant.

## 4. Discussion

Reactive oxygen species are crucial signaling molecules in various biological processes and are produced as a natural consequence of oxidative metabolism [27]. On the other hand, excessive ROS generation has been related to aging and several diseases, including chronic inflammation [28], diabetes [1], Alzheimer’s [29], and cancer [30]. The extract has been examined for its effect on ROS production and ROS scavenging activity in this context, following the determination of other phenol chemicals in GAAE. Then, on *ex vivo-in vitro* colon biopsy cultures, second research was set up to evaluate how GAAE worked against EC-LPS-induced inflammation. We used LO12-amplified chemiluminescence in the presence of HRP to investigate the impact of the GAAE against hydrogen peroxide (H_2_O_2_). The extract demonstrated good and quick H_2_O_2_ neutralizing action in a dose-dependent manner. Chemiluminescence is a technique with low background, and LO12 is a luminescent probe that is substantially more sensitive than luminal luminescence [31]. In actuality, H_2_O_2_ is produced by superoxide dismutase or spontaneous breakdown of superoxide [32]. Because H_2_O_2_ is not particularly polar, it can readily escape the phagosome. It will be in charge of oxidative damage and signaling in phagocytes and outside the cell [33]. H_2_O_2_ oxidizes a variety of biomolecules, causing their function to be lost and cellular function to be compromised [7]. Furthermore, H_2_O_2_ can cause permeabilization of lysosome membranes, most likely due to the generation of HO during the Fenton reaction in the lysosome, which promotes lipid peroxidation [31,34]. As a result, this was an intriguing starting point for testing GAAE’s influence on H_2_O_2_. Flow cytometry was used to investigate the effect of GAAE on H_2_O_2_. Heat inactivated yeasts tagged with DCFH2, and Alexa405 dies were utilized for the experiment. DCFH2 is oxidized to DCF in the presence of H_2_O_2_. The fluorescence generated by DCF was reduced dose-dependent after GAAE treatment. Alexa405 was treated with GAAE; it became less autofluorescent following laser excitation. The finding indicates that GAAE inhibits fluorescence emission. This interaction with fluorescence hides any possible H2O2 scavenging effect. As a result, the impact of GAAE on DCFH2-A405-yeast was investigated twice: once concurrently with H_2_O_2_ and again following DCF oxidation by H_2_O_2_. GAAE impacted DCF fluorescence in both trials, although to a smaller extent when DCF was pre-oxidized. This finding backs up GAAE’s fluorescence quenching impact. The difference between the two fluorescence rates was substantial at dosages of 250 ng/ml, 2.5 μg/ml, and 25 μg/ml, indicating an H_2_O_2_ neutralizing effect. The effect of GAAE on ROS-cell generation was examined second-time PLB-WT cells behave like neutrophils after differentiation and create reactive oxygen species when stimulated with PMA. The three-hour pretreatment of PLB with GAAE dramatically reduced DCF fluorescence-induced ROS generation in a dose-dependent manner. DCFH2 is a well-known probe that is thought to detect hydrogen peroxide in the presence of peroxidase. The non-fluorescent DCFH2 is oxidized, resulting in a brilliant fluorescent D [35]. For DCFH2, HOCI is a potent oxidant [24]. Its formation necessitates the presence of H_2_O_2_, and it is capable of attacking any oxidizable group [36]. Myeloperoxidase (MPO) is released when phagosomes and lysosomes fuse in active neutrophils. MPO may convert H_2_O_2_ to HOCI, which oxidizes DCFH2, and it can also serve as a peroxidase, catalyzing DCFH2 oxidation by H_2_O_2_ [37]. As a result, MPO plays a crucial role in determining the rate of DCFH2 oxidation [24,38]. GAAE may alter MPO activity or inhibit cell degranulation because it was employed as pre-treatment and rinsed before adding PMA, hence before ROS production. As a result, the extract can serve as an anti-inflammatory against phagocyte-induced inflammation in response to various stimuli. These findings encouraged researchers to investigate the effect of GAAE on human colon inflammation induced by EC-LPS.

In cultures of the normal colon, the extract was cultured for 24 hours with EC-LPS. According To Studies Published In The Literature, the EC-LPS produced gut inflammation in healthy rats [39] and human colon biopsies acquired from healthy volunteers [40] according to studies published in the literature. EC-LPS boosted the expression of NF-κB, which is involved in the production of proinflammatory genes such as IL-6 [41]. The MAPK and NF-κB signaling pathways are thought to be major intracellular molecular pathways involved in the inflammatory cascade response to EC-LPS stimulation in RAW264.7 cells [42], which is the case in our investigation. The primary inflammatory marker that increased in all colon cultures was COX-2. NF-κB promotes the expression of proinflammatory genes such as COX-2 and inducible nitric oxide synthase in inflammatory colons [43,44]. The proinflammatory expressions were affected by the GAAE co-treatment. On ICAM-1 and MAPK p38, there was no discernible impact.

On the other hand, GAAE has significantly reduced the transcription factor and the active key of the NF-κB inflammatory cascade. Furthermore, following GAAE therapy, COX-2, which is typically implicated in LPS-induced inflammation [44] was significantly reduced. GA is high in phytochemical substances that have anti-inflammatory properties and may help to reduce the effects of EC-LPS on the colon. The antioxidant and anti-inflammatory action of GAAE against ulcerative colitis exhibited *in vitro* and the reduction of proinflammatory markers in *ex vivo* may validate the antioxidant [45] and anti-inflammatory effect of GAAE against ulcerative colitis previously demonstrated in rats [15].

## 5. Conclusion

To summarize, the antioxidant properties demonstrated *in vitro* and the anti-inflammatory properties against proinflammatory markers in human colon biopsy cultures after the EC-LPS challenge suggest that GAAE could be a potential remedy to be considered for the biological treatment of various diseases involving inflammation and oxidative stress.

## Figures and Tables

**Table 1:** Characterization of GAAE extract by Liquid Chromatography-Electrospray Ionization Mass Spectrometry (LC-ESI-MS) analysis.

No^1^	Compounds^2^	Molecular formula	Molecular mass	[M-H]^−^ *m*/*z*	Retention time (min)	Content (μg/g extract)
1	Quinic acid	C_7_H_12_O_6_	192	191	2	2820.7
2	Protocatechuic acid	C_7_H_6_O_4_	154	153	6.9	133.6
3	Chlorogenic acid	C_16_H_18_O_9_	354	353	11.7	6.9
4	4-*O*-Caffeoylquinic acid	C_16_H_18_O_9_	354	353	11.8	7.14
5	Caffeic acid	C_9_H_8_O_4_	180	179	14.6	15.2
6	Syringic acid	C_9_H_10_O_5_	198	197	16.2	26.1
7	*p*-Coumaric acid	C_9_H_8_O_3_	164	163	21	659.4
8	Luteolin-7-*O*-glucoside	C_21_H_20_O_11_	448	447	24.8	1156.2
9	Rosmarinic acid	C_18_H_16_O_8_	360	359	26.2	140.7
10	Naringin	C_27_H_32_O_14_	580	579	26.4	229.9
11	Apigenin-7-*O*-glucoside	C_21_H_20_O_10_	432	431	27.2	14
12	*trans*-Cinnamic acid	C_9_H_8_O_2_	148	147	32	14720.4
13	Quercetin	C_15_H_10_O_7_	302	301	32.3	2.98
14	Kaempferol	C_15_H_10_O_6_	286	285	32.2	138.8
15	Naringenin	C_15_H_12_O_5_	272	271	34.2	1.7
16	Apigenin	C_15_H_10_O_5_	270	269	34.8	3.9
17	Luteolin	C_15_H_10_O_6_	286	285	35.2	1.6
18	Cirsiliol	C_17_H_14_O_7_	330	329	35.8	84.2
19	Cirsilneol	C_18_H_16_0_7_	380	343	38.9	0.56

## Abbreviations

GAAE: *Globularia alypum* aqueous extract
LC-ESI-MS: Liquid Chromatography-Electrospray Ionization Mass Spectrometry
H_2_O_2_: hydrogen peroxide
ROS: reactive oxygen species
HRP: horseradish peroxidase
MPO: Myeloperoxidase
PMA: Phorbol myristate acetate
EC-LPS: Escherichia coli-lipopolysaccharides
COX-2: cyclooxygenase 2
NF-κB: Nuclear factor-kappa B
ICAM-1: Intercellular Adhesion molecule 1
MAPK p38: Mitogen-activated protein kinases p38
